# Report on the Progress of Anatomy and Physiology, in the Year 1842-3

**Published:** 1844-02-24

**Authors:** James Paget

**Affiliations:** Lecturer on General and Morbid Anatomy and Physiology, and Warden of the Collegiate Establishment, at St. Bartholomew’s Hospital


### RECORD OF MEDICAL SCIENCE.

*Report on the Progress of Anatomy and Physiology, in the year* 1842-3. By James Paget, Lecturer on General and Morbid Anatomy and Physiology, and Warden of the Collegiate Establishment, at St. Bartholomew's Hospital.

This is the title of a very able paper contained in the British and Foreign Medical Review, for January, 1844. Under the belief that we cannot present to our readers anything better or more interesting, we extract a portion of this document, and shall continue to quote from it in future numbers, as space may allow, until the whole is given. It furnishes astonishing evidence of the talent and industry employed at the present day in the improvement of the branches of medical science which it embraces.

"The rule followed in this Report is that those works alone are noticed which were published between the 1st day of October, 1842, and the last of September, 1843;" and only those new things are noticed which have, in the author's opinion, been proved or rendered probable in the period and in the department which the report embraces.

Ed.

BLOOD.

Concerning the *general constitution* of the blood, MM. Andral and Gavarret, continuing with M. Delafond the observations already made on human blood, have examined that of many domestic animals, and have drawn these conclusions:—1. In the species examined, the principal constituents of the blood are the same, but the proportions of each vary. 2. The highest natural average quantity of fibrin is in the herbivora, the lowest in the carnivora; the energy of constitution has no constant influence on the increase of the proportion of fibrin. 3. Of the corpuscles the highest average proportions are found in the carnivora, the lowest in the herbivora; and in the same species there is always an increase proportionate to the energy of the constitution; and in sheep, to the improvement of the breed. 4. During the first day after birth the fibrin is in very small quantity; the corpuscles comparatively abundant. 5. During the last periods of gestation, both fibrin and corpuscles fall below the healthy proportion; after parturition they increase to more than that proportion. 6. The fibrin (in the domestic animals as well as in man) is always increased in the inflammatory state: the corpuscles are never directly influenced in it. 7. The water of the blood has its lowest average in the carnivora, its highest in the herbivora. 8. Dropsy does not supervene on alteration of the blood, unless from diminution of albumen; excess of water or decrease of corpuscles will not produce it.

*Coagulation.* Dr. Polli, from a long series of experiments on the influence of various gases on the coagulation of the blood, has shown how the discrepancy of previous experiments has depended on inattention to their details. All other conditions being the same, important differences as to time and mode of coagulation result from temporary exposure of the blood to the air, or from air remaining in the vessel, &c. Avoiding all these sources of fallacy, he found that coagulation takes place in pure oxygen or nitrogen, just as in the atmospheric air; but that the presence of carbonic acid always impedes coagulation. Carbonic acid is always given off in coagulation; and the greater the freedom with which it can be evolved, the more are the circumstances favorable to coagulation. The more carbonic acid the blood itself contains the slower is the coagulation, and the greater the chance of a buffy coat being formed; and a buffy coat being formed, without froth and over a dark clot, is always a sign of the blood being surcharged with carbonic acid.

*Buffy coat.* The exact mode of the formation of the buffy coat has been well illustrated by Mr. Wharton Jones, whose observations on the blood I can in nearly all points confirm. He ascribes it, as Nasse and others did, chiefly to the tendency of the blood-corpuscles to arrange themselves rapidly in rolls (like rolls of coins,) which form a wide-meshed net-work, (as seen in a single layer under the microscope,) or a kind of spongework when they are kept in mass. In the former case the liquor sanguinis coagulates within the meshes of the rows of the corpuscles, and hence the distinctly mottled aspect of the layer when coagulated as well as when first drawn; in the latter case the spongework formed by the rows of corpuscles contracts and squeezes out the liquor sanguinis, and permits the greater specific gravity of the corpuscles to come into play, so that they sink quickly, and the liquor sanguinis floats to the top and coagulates in a distinct layer. In both cases the pale corpuscles remain with the separated liquor sanguinis and are imbedded in its white coagulum.* The attraction for each other by which the corpuscles tend to unite in rolls, is so remarkably increased in the state of the blood in which a buffy coat is formed, that the early or instantaneous occurrence of this arrangement of them, as seen by the microscope in a single drop of blood, affords all the evidence which could be derived from the formation of a buffy coat on a large quantity.

*Corpuscles.* On the size of the blood-corpuscles, Mr. Gulliver has added to his former copious observations the measurements of those of many more species of mammalia and birds; and on their structure he has adduced, from a comparison of the effects of water, acetic, and muriatic acids, and other agents upon the blood-corpuscles of mammalia and birds, further evidence for the belief that those of the former do not contain nuclei; or, rather, that although their central may differ from their peripheral matter, the former does not stand in the relation of a nucleus to the latter, except in the blood-corpuscles of embryos. A chief point in the evidence is, that in the corpuscles of birds, small as they are, a nucleus may be demonstrated; and so plainly, that if such an one existed in the mammal's corpuscle, it could not escape notice when similarly searched for. Mr. Wharton Jones also denies the existence of the nucleus in these corpuscles: and shows that the particles which are supposed to be nuclei exposed by the action of acetic acid on the corpuscles of mammalia, are only portions of albumen coagulated by the acid, such as may be produced by adding acid to liquor sanguinis or serum. At the same time it is

* The tendency of the pale corpuscles to keep with the liquor sanguinis is also shown by Mr. Gulliver, in his observations that fibrin obtained by washing, even when transparent and quite colorless, contains these corpuscles in great numbers. (Lond. and Edinb. Phil. Mag., Aug. 1842.) I have found that films of liquor sanguinis which have coagulated on healthy blood drawn under oil, are composed entirely of pale corpuscles held together like the cells of a tesselated epithelium.

not to be supposed that the corpuscle is homogeneous throughout; the central substance is evidently different from the peripheral, and Mr. Jones' opinion seems very probable, that the appearance of a nucleus depends on the walls of the corpuscle being thick, consisting of two layers, the outer transparent, colourless, and resisting; the inner softer and less resisting. Between the two, or perhaps in the inner layer, the colouring matter is contained. The existence of a true nucleus in the corpuscles of the lower vertebrata is not by this rendered doubtful; and Mr. Addison has shown, in the use of *liquor potassæ*, a good method of demonstrating it in the corpuscles of the frog.

Many interesting observations have been made by both Mr. Wharton Jones and Dr. Carpenter, in support of the view that some of the corpuscles of the blood are a kind of "floating gland cells." The former believes that the office of the red corpuscles is to convert albumen into fibrin, elaborating it in their interior, and then, after the manner of gland cells, dissolving and discharging their contents. The latter thinks it more probable that this office of converting the chemical compound into the organizable principle, is discharged by the pale corpuscles, and that the red corpuscles are the chief carriers of oxygen and carbonic acid. The arguments of each may be found in the last October and January numbers of this Journal. Which ever view be most true, it may be received as highly probable, that the corpuscles in the blood are the agents by which, with the aid of the oxygen imbibed in the lungs, the liquid portion is brought into a state fit for the nutrition of the tissues, and that this is their chief purpose.

Mr. Macleod has described the development of the blood corpuscles in the chick, dividing it into three stages. At first there are no particles in the blood except minute dark spherical granules. These gradually enlarge and become clear in their centres; but when they have arrived at double of their original size, the central part of each becomes dull and then distinctly granular, while the border becomes defined, smooth and clear. This completes the first stage; after which, in the second, the central granules disappear as if they had merged into one central clear nucleus, from which the external portion slowly separates, not at one side only, but all round. In this stage the corpuscle remains circular; but it becomes flatter, both on its surfaces and its edges, and a concave furrow forms between its outer border and the border of the nucleus. At the same time also it acquires colour apparently by the accumulation of colouring matter in the space between the nucleus and capsule. In the third stage, the corpuscle assumes the oval shape; first, one side of both the cell and nucleus gradually stretching out, and then the other, so that every part of the corpuscle becomes narrower except the middle. Coincidently with these changes, the furrow around the nucleus disappears, and the sharp edges of the borders are smoothly rounded off.

Mr. Macleod believes that all those changes are effected by a power dwelling in the granules, each of which developes itself into a cell. He has never seen any congregation of granules to form a cell, nor any multiplication of granules within the once formed nucleus, nor any opening in the centre of the nucleus, nor any escape of corpuscles from it.

#### FIBRO-CELLULAR TISSUE.

Dr. Todd and Mr. Bowman have thrown doubt on the received opinion concerning the structure of the fibro-cellular tissue. They regard it not as consisting of bundles of parallel filaments of definite size and structure, but as a substance which has a tendency to split up almost *ad infinitum* in the longitudinal direction, and which has a filamentous appearance from streaks and creasings on its surface. [In small hard tendons, such as those of insects, this description may hold; they are compact and nearly homogeneous bands, with scarcely any appearance of a filamentous composition; but in the looser varieties of fibro-cellular tissue I cannot doubt the existence of bundles of distinct filaments.]

A few facts concerning the anatomy of tendons may be collected from the long discussions on tenotomy at the Parisian Academy of Medicine.

#### MUSCULAR TISSUE.

I have lately found a mode of attachment of the ultimate fibres of muscles to their tendons which has not yet, I think, been made known. It may be distinctly seen in the muscle torn out from the leg of a fly. Each of three tendons, which are planted in the proximal end of the last but one articulation of the leg, runs in a long straight and flat band up the interior of the next superior division of the limb, and receives on each of its edges the broad and somewhat rounded bases of the muscular fibres. These are arranged in a penniform manner, the base of each fibre on one side of the tendon corresponding to the halves of the bases of two adjacent fibres on the opposite side, like the leaflets of the pteris and some other ferns. The fibres are flat, and their extremities, instead of being ensheathed in the tendinous tissue, only adhere to the border of the tendon, and receive on their outer edges one or two finer tendinous filaments, as if for greater fixity.

Dr. Remak has found that portions of the diaphragm, the heart, and the muscular walls of the larger vessels of many animals of all classes, will continue spontaneously contracting for as many as forty-eight hours after death. He has also pointed out their several modes of contraction, (?) which he distinguishes as creeping, undulating, peristaltic, and serpentine or zig-zag.

*Rigor Mortis.* An ingenious paper has been published by Ernst Bruecker, to prove that the rigor mortis is due to the coagulation of the fibrin which is effused from the blood vessels in the liquor sanguinis for the nutrition of the tissues, (especially of the muscles,) but which at the time of death has not yet been assimilated. The paper is chiefly important for the numerous analogies which it points out between the coagulation and subsequent changes of the fibrin and the contraction of the muscles; analogies of which Mr. Hunter had already illustrated many, and some of the most important. But the explanation of the rigor mortis is rendered improbable by the observations of Mr. Bowman, which I can fully confirm, and which prove that the muscle is rigid because its fibres, or parts of them, are contracted, and contracted in the same manner as during life. And some examinations which I have made of the rigor mortis in the involuntary muscles, afford equally strong evidence of its being due in them also to the muscular contraction. I believe that *all involuntary muscles* pass into the continued and fixed contraction of the rigor mortis as soon as they cease to be irritable, and to contract during ordinary stimuli. This may be seen distinctly in many arteries, as well as in the digestive canal and urinary bladder; but the best examples are presented in the hearts of recently slain animals, or of men examined soon after apparent death.* As soon as they cease to be irritable, the walls of all their cavities, previously flaccid, gradually become firm and hard, draw in towards the cavity of the heart, and reduce or completely close the cavities. The heart thus rigid has almost exactly the form and other external characters of the heart when actively contracted during life. It is evident that this form, produced as it is by a drawing up towards certain fixed points of attachment of the muscular fibres, could not be acquired by the mere coagulation of either blood or liquor sanguinis within the tissue of the heart; and the only difficulty in believing that the rigidity of the heart and other muscles is due to a contraction comparable with that which occurs during more active life, must be from the seeming improbability that any tissue should maintain a vital contraction so long after apparent death as during the continuance of the rigor mortis. This difficulty, however, which would in any circumstances be more apparent than real, is almost removed by the facts already quoted from Remak.

* Hearts in the state of rigor mortis are those commonly described as affected by concentric hypertrophy. Dr. George Budd proved some years ago that this appearance of an increased thickness of the walls with diminution of the cavities could not be due to disease of the heart; (Medico-Chirurg. Trans., vol. xxi. p. 296 ;) and I may add to the evidence which he adduced, (and which ought to have been taken as decisive of the question,) that in every instance the hearts of healthy oxen become affected with an extreme degree of *concentric hypertrophy* within an hour after they are slaughtered. I have little doubt also that the hearts of all persons pass into a similar state within a few hours after apparent death; certainly a large majority of the hearts examined within the first ten hours have more or less the appearance of being contracted, and many retain the appearance for twenty-four or thirty-six hours. In the majority, relaxation and flaccidity return after the first day. But the rules as to time by which the rigor mortis is regulated in both the voluntary and the involuntary muscles have yet to be studied.

